# Cultural Engagement and Hospital-Treated Mental Disorders in Older Adults

**DOI:** 10.1001/jamanetworkopen.2026.39041

**Published:** 2026-10-14

**Authors:** Pei Qin, Andrew Steptoe, Daisy Fancourt

**Affiliations:** 1 Department of Behavioral Science and Health, University College London, London, United Kingdom

## Abstract

**Importance:**

Cultural engagement is associated longitudinally with better mental health and reduced depression incidence, but evidence has largely relied on self-reported symptoms and diagnoses, leaving uncertainty about clinically recorded disorders, and residual confounding remains a concern.

**Objective:**

To examine whether cultural engagement is associated with incident hospital-treated mental disorders.

**Design, Setting, and Participants:**

This prospective cohort study included adults aged 50 years and older from the English Longitudinal Study of Aging (wave 4, May 2008 to July 2009), with follow-up through March 2024. Data were analyzed from November 2025 to March 2026.

**Exposures:**

Frequency of engagement with any of the receptive arts activities (ie, watching or consuming performances or exhibitions) was categorized as never, infrequent (less than once a year up to once or twice a year), or frequent (every few months up to monthly or more).

**Main Outcomes and Measures:**

Participant records were linked to diagnostic codes in Hospital Episode Statistics and mortality records with follow-up of up to 20 years. The primary outcomes were mental disorders overall and 4 main mental disorders (dementia, substance misuse, mood disorders, and neurotic disorders). Cox proportional hazard regression models were used to estimate the hazard ratios (HRs) and 95% CIs, with a false discovery rate (FDR) approach for significance. To further probe causality, eye disease, ear disease, and traumatic brain injury—which share similar sociodemographic profiles to mental disorders—were prespecified as negative control outcomes.

**Results:**

This study included 8274 adults (mean [SD] age, 65.4 [9.6] years; 4672 female [55.2%]). After fully adjusting for sociodemographic, lifestyle, and social factors and multiple testing, frequent cultural engagement was associated with lower risk of any mental disorders (HR, 0.71; 95% CI, 0.62-0.82; *P*_FDR_ < .001), dementia (HR, 0.71; 95% CI, 0.56-0.89; FDR-adjusted *P* = .01), substance misuse (HR, 0.75; 95% CI, 0.59-0.95; FDR-adjusted *P* = .04), and mood disorders (HR, 0.73; 95% CI, 0.56-0.95; FDR-adjusted *P* = .04), but not neurotic disorders. Associations persisted after excluding early incident cases and adjusting for baseline depressive symptoms and cognition and showed robustness to unmeasured confounders. Cultural engagement was not associated with any negative control outcomes.

**Conclusions and Relevance:**

In this nationally representative cohort of older adults, cultural engagement was associated with a lower hazard of subsequent hospital-treated mental disorders, with particularly consistent associations for dementia, substance misuse, and mood disorders. These findings provide triangulated statistical data to suggest that cultural engagement is associated with reduced risk of several clinically recorded mental disorders and support further testing of cultural engagement as a population mental health strategy.

## Key Points

**Question:**

Is cultural engagement associated with incident hospital-treated mental disorders?

**Findings:**

This cohort study of 8274 adults found that frequent cultural engagement was associated with lower risk of mental disorders overall, as well as specific disorders including dementia, substance misuse, and mood disorders. Associations persisted in sensitivity analyses addressing prodromal bias and unmeasured confounding.

**Meaning:**

These findings provide triangulated statistical data to suggest that cultural engagement is associated with reduced risk of several clinically recorded mental disorders. They align with findings from experimental studies suggesting the potential value of cultural engagement as a population mental health strategy.

## Teaser

This cohort study examines associations of cultural engagement with incident hospital-treated mental disorders including dementia, substance misuse, mood disorders, and neurotic disorders among adults aged 50 years and older in England.

## Introduction

The arts encompass a diverse range of human practices relating to the production or experience of human creativity and imagination. Arts engagement can include actively participating in the arts (eg, dance, music, and crafts) and attending cultural events (eg, going to live music events, museums, galleries, theater performances, and the cinema; hereafter referred to as *cultural engagement*).^1^ There is increasing recognition that arts participation and cultural engagement are health-promoting behaviors that impact a wide range of health outcomes.^2^

Epidemiological research on cultural engagement has most commonly focused on outcomes related to mental health and brain health. Both cross-sectional and longitudinal studies have demonstrated that cultural engagement is associated with better mental well-being,^3,4,5,6^ lower levels of self-reported depressive symptoms and anxiety,^6,7,8,9,10^ and better preservation of cognition.^11,12^ Some studies have also reported decreased risk of incident mental disorders. For example, one study using data from the English Longitudinal Study of Aging (ELSA) found a dose-response association of frequency of cultural engagement with reduced risk of incident depression in older adults over the following decade, independent of sociodemographic, health-related and social confounders.^7^ Another longitudinal study in the Danish general population found associations of low levels cultural engagement with both new onset and persistent depression.^10^ Reduced incidence of and a longer time to dementia was found among people with more frequent cultural engagement.^13^ However, there remain a number of gaps in the literature.

First, previous research is limited to self-reported diagnoses of mental disorders, which could be subject to recall or social desirability biases, or to validated symptom scales that may not capture diagnoses that have been made but are now well-managed with medication or psychotherapy. We lack studies that use objective diagnostic data from electronic patient health records. Second, studies on incidence have focused exclusively on depression to date. Both theoretical work positioning arts within a transdiagnostic, process-based model of mental health, and experimental work demonstrating causal effects on a wide range of psychiatric conditions suggest cultural engagement may have a preventive effect on a broader range of mental disorders.^14,15,16^ Third, cultural engagement includes behaviors that typically show sociodemographic patterning. Previous studies have used a variety of statistical methods to explore independence of effects, including conditioning on a broad range of identified confounders, applying fixed-effects methods that consider time-varying and unmeasured confounders, and using instrumental variable approaches. However, confounding remains an important consideration, and triangulation of findings from a variety of different statistical methods is important to strengthen causal certainty. Approaches such as negative control outcomes have yet to be applied to considerations of cultural engagement and mental disorders.^17^ Finally, there have been concerns that previously reported associations of arts participation and cultural engagement with incident mental disorders could be byproducts of prodromal effects, with early symptoms leading to a progressive reduction in leisure activities.^18^ There is limited longitudinal data extending beyond 10-year follow-up periods, which could help to disentangle reverse causal effects.

To fill in these knowledge gaps, the present study aimed to investigate the association of cultural engagement with hospital-treated mental disorders over a follow-up period of up to 20 years by leveraging nationally representative data in the ELSA study linked to UK national Hospital Episode Statistics records of conditions requiring hospital treatment and adopting a negative control analysis. We also tested the persistence of findings when exploring further methodological issues such as reverse causality, measurement error, and residual confounding.

## Methods

### Study Design and Sample

This cohort study was based on ELSA, a nationally representative panel study of participants aged 50 years or older living in private households in England. ELSA commenced in 2002, with follow-up every 2 years.^19^ In this study, we analyzed data at wave 4 (May 2008 to July 2009) as our baseline because this wave had the largest number of participants for the measurement of cultural engagement and covariates considered. ELSA received ethical approval from the National Research Ethics Service, and written informed consent was obtained from study participants. This study followed the Strengthening the Reporting of Observational Studies in Epidemiology (STROBE) reporting guideline.

Participants were eligible for inclusion if they had given consent to the data linkage and were aged 50 years or older and had complete data on cultural engagement at baseline (eFigure 1 in Supplement 1). Sample size was then allowed to vary for each *International Statistical Classification of Diseases and Related Health Problems, Tenth Revision (ICD-10)* code to maximize usage of the available data by only excluding those who had corresponding mental health conditions at or before baseline.

### Measurement of Cultural Engagement

We focused specifically on receptive arts participation activities (ie, watching or consuming performances or exhibitions). Participants were asked about the frequency of visits to (1) the cinema; (2) art galleries, exhibitions or museums; and (3) theater, concerts, or opera. Each cultural engagement item was assessed on a 5-point scale from 0 (never), to 5 (≥2 times a month). Frequency of engagement with any of these activities was categorized as never, infrequent (less than once a year to once or twice a year), or frequent (every few months to monthly or more), because the highest-frequency categories contained relatively few participants, thereby improving the stability of statistical estimates and facilitating interpretation.

### Measurement of Outcomes

We ascertained mental disorders during follow up on the basis of hospital episode statistics and causes of death up to March 2024, using *ICD-10* codes (eMethods and eTable 1 in Supplement 1). We focused on mental disorders overall and 4 main mental disorders, including dementia (F00-F03, G30, and G31), substance misuse (F10-F19), mood disorders (F30-F39), and neurotic disorders (F40-F48). Additionally, we applied a negative control design to strengthen understanding of whether the association was likely to be causal or whether it was a result of unmeasured or residual confounding.^20^ For our negative controls, we selected eye diseases, ear diseases, and traumatic brain injuries because these have been demonstrated to have similar demographic and socioeconomic patterning to mental disorders in prior publications.^21,22,23^ For each health outcome, participants with that specific health condition at or before baseline ascertained from the linked health registry data were excluded from the analysis of incident hospital-treated conditions.

### Covariates

Variables considered likely to confound the associations of cultural engagement with health outcomes were measured at baseline (wave 4).^24^ Details can be found in the eMethods and eFigure 2 in Supplement 1. Ethnicity data were collected via self-reported questionnaire and were included as a covariate because cultural engagement may be associate with both patterns of cultural engagement and the risk of mental disorders; ethnicity was categorized as White (defined as White British, White Irish, and any ethnic group with a White racial background) and any ethnicity other than White (which included multiethnic groups, Black and Black British, Asian and Asian British, and any other ethnic group whose racial background is not White).

### Statistical Analysis

We performed a complete case analysis. The Kaplan-Meier method was used to estimate the cumulative incidence of mental disorders and negative control diseases, and differences in curves were tested by log-rank test. Separate Cox proportional hazard regression models were used to estimate the hazard ratios (HRs) and 95% CIs. Proportional hazard assumptions were tested using Schoenfeld residuals tests. Variation inflation factors were calculated to test the presence of multicollinearity among the variables. To visualize changes in risk and relative magnitude of associations across outcomes, Manhattan plots were used to show overall associations of cultural engagement with mental disorders over the follow-up period. We performed a series of sensitivity analyses to assess potential biases (eMethods in Supplement 1).

All analyses were conducted using R version 4.3.2 (R Project for Statistical Computing) from from November 2025 to March 2026. Two-sided *P* values were calculated and then corrected for multiple testing by using the Benjamini-Hochberg false discovery rate (FDR) approach.^25^ Findings were considered statistically significant if the FDR-adjusted *P* value was less than .05.

## Results

Table 1 presents the demographics of this analytic sample. Of the 8464 adults included, 4672 (55.2%) were female, 8728 (97.8%) were of White ethnicity, 186 (2.2%) were an ethnicity other than White, and the sample had a mean (SD) age of 65.4 (9.6) years at baseline.

**Table 1.** Baseline Characteristics of Participants Overall and by Cultural Engagement

Characteristic	Participants, No. (%)				*P* value
	Overall (N = 8464)	Cultural engagement			
		Never (n = 1566)	Infrequent (n = 3582)	Frequent (n = 3316)	
Age, mean (SD), y	65.4 (9.6)	69.9 (10.9)	65.0 (9.4)	63.6 (8.5)	<.001
Sex					
Female	4672 (55.2)	816 (52.1)	1946 (54.3)	1910 (57.6)	.001
Male	3792 (44.8)	750 (47.9)	1636 (45.7)	1406 (42.4)	
Education group					
No educational qualifications^a^	2138 (25.3)	822 (52.7)	888 (24.9)	428 (12.9)	<.001
Basic qualifications	2715 (32.2)	464 (29.7)	1283 (35.9)	968 (29.2)	
High school qualifications	692 (8.2)	72 (4.6)	308 (8.6)	312 (9.4)	
College degree or higher	2899 (34.3)	203 (13.0)	1094 (30.6)	1602 (48.4)	
Race and ethnicity^b^					
White	8278 (97.8)	1523 (97.3)	3503 (97.8)	3252 (98.1)	.17
Other	186 (2.2)	43 (2.7)	79 (2.2)	64 (1.9)	
Marital status: married or cohabiting	5915 (69.9)	903 (57.7)	2612 (72.9)	2400 (72.4)	<.001
Wealth quintile					
1 (Lowest)	1659 (20.0)	586 (38.1)	685 (19.5)	388 (12.0)	<.001
2	1658 (20.0)	406 (26.4)	761 (21.6)	491 (15.2)	
3	1658 (20.0)	254 (16.5)	755 (21.4)	649 (20.1)	
4	1658 (20.0)	192 (12.5)	695 (19.7)	771 (23.9)	
5 (Highest)	1658 (20.0)	102 (6.6)	625 (17.8)	931 (28.8)	
Employment: working full or part time	2887 (35.9)	252 (16.6)	1260 (36.9)	1375 (44.3)	<.001
Occupational status					
Managerial and professional	2927 (35.7)	252 (16.7)	1158 (33.4)	1517 (46.9)	<.001
Intermediate	1209 (14.7)	162 (10.7)	525 (15.2)	522 (16.2)	
Small employers and self-employed	951 (11.6)	167 (11.0)	416 (12.0)	368 (11.4)	
Lower supervisory and technical	737 (9.0)	198 (13.1)	351 (10.1)	188 (5.8)	
Semiroutine	2383 (29.0)	734 (48.5)	1012 (29.2)	637 (19.7)	
Smoking status					
Never	3383 (40.1)	478 (30.6)	1400 (39.2)	1505 (45.5)	<.001
Ever	3969 (47.0)	718 (45.9)	1687 (47.2)	1564 (47.3)	
Current	1094 (13.0)	367 (23.5)	487 (13.6)	240 (7.3)	
Alcohol: ≥5 d/wk	1941 (23.1)	260 (16.7)	776 (21.8)	905 (27.4)	<.001
Sedentary	645 (7.6)	277 (17.7)	241 (6.7)	127 (3.8)	<.001
Social isolated	534 (6.3)	163 (10.4)	201 (5.6)	170 (5.1)	<.001
No community activities	2310 (28.3)	734 (51.2)	1007 (29.1)	569 (17.5)	<.001
Live alone	1855 (21.9)	516 (33.0)	696 (19.4)	643 (19.4)	<.001

^a^ Basic qualifications refers to General Certificate of Education (GCE) O-levels or national vocational qualification (NVQ) 2, while high school qualifications refer to NVQ 3 or GCE A-levels.

^b^ White included White British, White Irish, and any White ethnicity not otherwise specified and Other included multiethnic, Black and Black British, Asian and Asian British, and any other ethnic group whose race is not White.

### Cultural Engagement

Table 1 presents the distribution of cultural engagement by demographic characteristics. Of the whole cohort, 1566 (18.5%) reported never engaging in any cultural activities, 3582 (42.3%) engaged infrequently (less than once a year up to twice a year), and 3316 (39.2%) engaged frequently (every few months or more). Compared with the never engaged group, those who engaged frequently were more likely to be younger, female, married or cohabiting, work full or part time, be employed as managerial and professional or intermediate area, and have higher education qualifications and wealth. They were also less likely to smoke, drink more than 5 days per week, or be socially isolated, and they were more physically active and engaged in more community activities.

### Cultural Engagement and Incidence of Mental Disorders

Figure 1 shows Kaplan-Meier curves of the incidence of mental disorders by cultural engagement frequency. In Cox models adjusted just for age and sex, cultural engagement was associated with a lower hazard of developing all mental disorders (Table 2). In fully adjusted models, this association persisted for overall mental disorders (frequent cultural engagement: HR, 0.71; 95% CI, 0.62-0.82; *P*_FDR_ < .001), dementia (frequent cultural engagement: HR, 0.71; 95% CI, 0.56-0.89, FDR-adjusted *P* = .01), substance misuse (frequent cultural engagement: HR, 0.75; 95% CI, 0.59-0.95, FDR-adjusted *P* = .04), and mood disorders (frequent cultural engagement: HR, 0.73; 95% CI, 0.56-0.95; FDR-adjusted *P* = .04). For neurotic disorders, the association was not significant after considering health-related behaviors. Manhattan plots in Figure 2 show the fully adjusted models of incidence over the follow-up period for overall mental disorders and the specific diagnostic categories.

**Figure 1.**
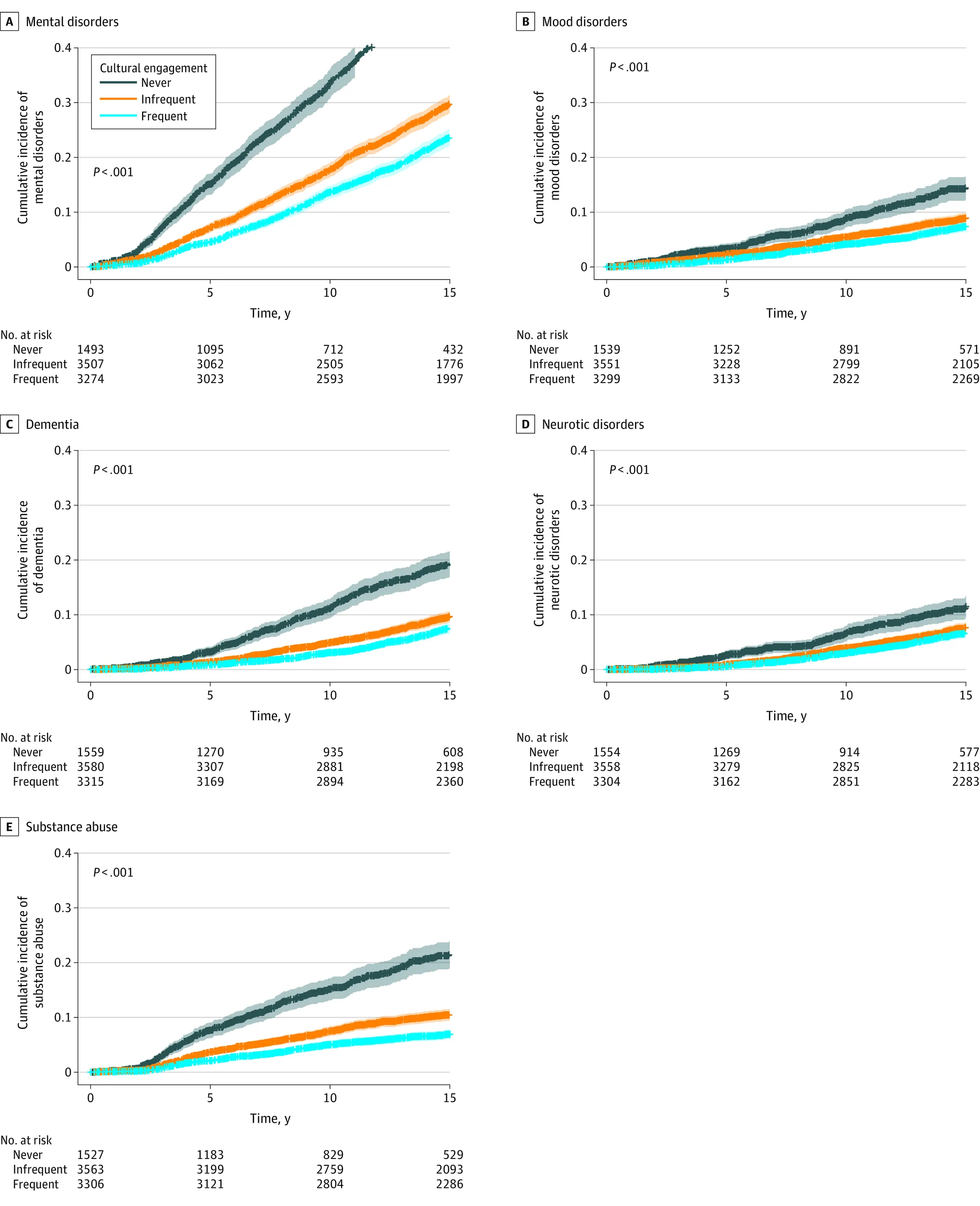
Line Graphs of Cumulative Incidence for Disease Categories Among People With Different Cultural Engagement Groups The reference group for cultural engagement was never. Shaded areas represent 95% CIs.

**Table 2.** Association of Cultural Engagement With Mental Disorders in the Different Adjustment Models^a^

Diseases by cultural engagement category	Model 0^b^			Model 1^c^			Model 2^d^			Model 3^e^		
	HR (95% CI)	*P* value	FDR-adjusted *P* value	HR (95% CI)	*P* value	FDR-adjusted *P* value	HR (95% CI)	*P* value	FDR-adjusted *P* value	HR (95% CI)	*P* value	FDR-adjusted *P* value
Overall mental disorders												
Infrequent	0.61 (0.55-0.67)	<.001	<.001	0.72 (0.64-0.81)	<.001	<.001	0.78 (0.70-0.88)	<.001	<.001	0.77 (0.69-0.87)	<.001	<.001
Frequent	0.48 (0.43-0.53)	<.001	<.001	0.61 (0.54-0.69)	<.001	<.001	0.73 (0.64-0.83)	<.001	<.001	0.71 (0.62-0.82)	<.001	<.001
Dementia												
Infrequent	0.70 (0.58-0.83)	<.001	<.001	0.73 (0.60-0.88)	.001	.004	0.75 (0.62-0.92)	.004	.01	0.79 (0.65-0.97)	.03	.05
Frequent	0.61 (0.50-0.74)	<.001	<.001	0.65 (0.53-0.81)	<.001	<.001	0.69 (0.55-0.86)	.001	.003	0.71 (0.56-0.89)	.004	.01
Substance misuse												
Infrequent	0.44 (0.37-0.52)	<.001	<.001	0.63 (0.52-0.76)	<.001	<.001	0.77 (0.64-0.93)	.008	.02	0.77 (0.63-0.93)	.008	.02
Frequent	0.28 (0.23-0.34)	<.001	<.001	0.46 (0.37-0.57)	<.001	<.001	0.78 (0.62-0.98)	.03	.07	0.75 (0.59-0.95)	.02	.04
Mood disorders												
Infrequent	0.62 (0.51-0.75)	<.001	<.001	0.76 (0.61-0.94)	.01	.03	0.80 (0.64-0.99)	.04	.08	0.79 (0.63-1.00)	.048	.09
Frequent	0.49 (0.40-0.61)	<.001	<.001	0.69 (0.54-0.88)	.003	.009	0.76 (0.59-0.97)	.03	.06	0.73 (0.56-0.95)	.02	.04
Neurotic disorders												
Infrequent	0.66 (0.53-0.82)	<.001	.001	0.79 (0.62-1.01)	.06	.11	0.84 (0.65-1.07)	.16	.22	0.84 (0.65-1.09)	.20	.26
Frequent	0.55 (0.44-0.69)	<.001	<.001	0.75 (0.58-0.98)	.04	.07	0.80 (0.61-1.06)	.12	.19	0.81 (0.61-1.08)	.15	.22
Negative control diseases												
Eye diseases												
Infrequent	0.91 (0.81-1.03)	.13	.20	0.95 (0.83-1.07)	.39	.44	0.94 (0.83-1.07)	.32	.38	0.94 (0.82-1.07)	.36	.42
Frequent	0.91 (0.81-1.02)	.12	.19	1.01 (0.88-1.15)	.91	.94	1.00 (0.87-1.15)	.97	.965	1.00 (0.86-1.15)	.95	.97
Ear diseases												
Infrequent	0.69 (0.41-1.17)	.17	.24	0.87 (0.48-1.55)	.63	.67	0.87 (0.48-1.56)	.63	.67	0.95 (0.51-1.77)	.87	.91
Frequent	0.58 (0.33-1.01)	.05	.09	0.71 (0.37-1.36)	.30	.36	0.70 (0.36-1.37)	.30	.36	0.81 (0.40-1.63)	.56	.62
Traumatic brain injury												
Infrequent	0.82 (0.63-1.08)	.16	.23	0.77 (0.58-1.03)	.08	.13	0.78 (0.59-1.05)	.10	.17	0.79 (0.58-1.07)	.13	.19
Frequent	0.88 (0.67-1.16)	.37	.43	0.82 (0.60-1.11)	.20	.26	0.84 (0.62-1.15)	.28	.36	0.82 (0.59-1.13)	.22	.29

Abbreviations: FDR, false discovery rate; HR, hazard ratio.

^a^ This table showed the risk estimates compared with the never engaged group.

^b^ Model 0 adjusted for age and sex.

^c^ Model 1 further adjusted for sociodemographic and economic factors (ethnicity, education, marital status, wealth, employment, and occupational status).

^d^ Model 2 further adjusted for lifestyle factors (smoking, alcohol drinking, and sedentary behavior).

^e^ Model 3 further adjusted for social covariates (living alone, social isolation, and engagement in other community activities).

**Figure 2.**
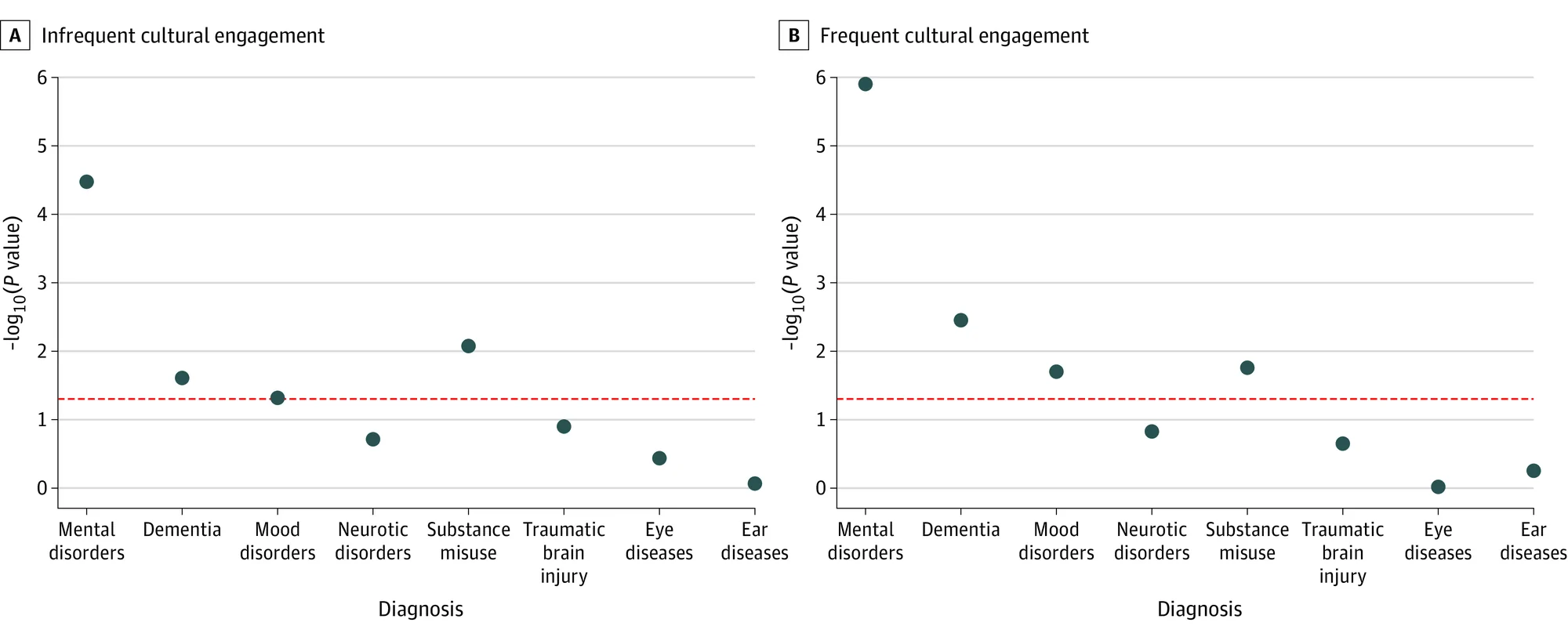
Manhattan Plots of the Association of Cultural Engagement With Mental Disorders The x-axis represents the disease categorical group, and the y-axis represents the negative log (10) of the *P* value for the Cox hazard regression model between frequent cultural engagement and disease outcomes. The red dashed horizontal line shows the *P* value of .05 (false discovery rate correction for multiple testing); dots above the line indicate a significant inverse association with cultural engagement. The model was adjusted for age, sex, ethnicity, education, marital status, wealth, employment, occupational status, lifestyle (smoking, alcohol drinking, sedentary behavior), and social covariates (living alone, social isolation, and other community activities).

### Persistent Latent Socioeconomic Confounding

We applied negative control outcomes to test whether the association could be the result of unmeasured or residual confounding. Across the follow-up period (maximum, 15.8 years), incidence of overall mental disorders were 28.1% (2258 cases), similar to the incidence of 29.5% (2259 cases) reported for eye diseases (eTable 2 in Supplement 1). Within each specific mental disorder diagnosis, incidences ranged from 6.9% (560 cases) for neurotic disorders to 9.7% (790 cases) for substance misuse, similar to the incidence rate of 4.6% (386 cases) for traumatic brain injuries, and a little higher than the incidence rate of 1.1% (92 cases) reported for ear diseases. The outcomes of mental disorders and the negative control outcomes had similar patterns of associative factors (eFigure 3 in Supplement 1). Thus, the negative controls were deemed appropriate comparison outcomes.

We leveraged the negative control outcomes and looked for similar associations with cultural engagement. No associations were observed for the negative control outcomes in the models just adjusted for age and sex, nor across any of the confounding models (Table 2 and Figure 2).

### Reverse Causality and Measurement Error

To test whether results were heavily influenced by reverse causality, such as people with imminent diagnoses already experiencing prodromal symptoms that could influence their cultural engagement, we undertook 2 further sets of analyses. First, we excluded those with diagnoses shortly after baseline, which had no impact on the results (eTable 3 in Supplement 1). Second, we additionally adjusted the model for overall mental disorders for baseline depressive symptoms and cognitive functioning and negative control outcomes. The association remained significant (eTable 4 in Supplement 1). The results of the index that simultaneously considered both frequency and diversity of engagement were broadly aligned, although there was a smaller-magnitude dose-response association for dementia (eTable 5 in Supplement 1).

### Robustness of Findings to Further Unmeasured Confounding and Longer Follow-Up

For unmeasured confounding,^26^ the E-values for the point estimates for frequent cultural engagement ranged from 1.77 to 2.17 (eTable 6 in Supplement 1). For comparison, in the overall mental disorder outcome, the confounder smoking status had the largest magnitude of association with the outcome (risk ratio, 4.62; 95% CI, 4.04-5.27) for current smoking, but this was something of an outlier. The confounder with the second largest magnitude of association was sedentary behaviors (risk ratio,  1.28; 95% CI, 1.09-1.49). These results indicate that an additional confounder that exceeds the association observed for every other measured confounder currently included in the model would be needed to explain away the observed association entirely.

The sensitivity analysis using wave 2 as baseline showed consistently significant associations, and cultural engagement was also found to be associated with neurotic disorders (eTable 7 in Supplement 1). To test alternative models, we additionally applied survey weights, but this had no material effect on the results (eTable 8 in Supplement 1). Further, the findings from the Fine-Gray competing-risk analyses were mostly consistent with those from the primary analyses, except for no association for dementia in the competing-risk analysis (eTable 9 in Supplement 1).

## Discussion

In this nationally representative cohort of older adults linked to Hospital Episode Statistics, cultural engagement was associated with a lower hazard of subsequent hospital-treated mental disorders, with particularly consistent associations for mood disorders, dementia, and substance misuse. These findings provide important advances on previous literature reporting preventive effects of arts and cultural engagement. First, they move beyond self-reported diagnoses and symptom scales by using objective clinical diagnoses in electronic patient health records. Second, they highlight that cultural engagement may be associated with risk across multiple diagnostic groupings. Third, the combination of long follow-up, nested confounder adjustment, negative control outcomes, and sensitivity analyses provides a stronger causal triangulation framework than has typically been applied. Notably, the magnitude of protective associations identified is comparable to other established behavioral determinants of mental health reported previously, such as physical activity, where prospective meta-analyses have reported 17% to 25% lower risk of incident depression among more active individuals.^27,28^ While these are indirect comparisons with the results presented here, and thus need to be treated with appropriate caution, they reinforce the potential salience of cultural engagement as a health-promoting behavior.

The findings presented are theoretically consistent with transdiagnostic models of mental health, which posit that diverse psychiatric conditions arise from perturbations in shared underlying processes, such as affect regulation, cognitive flexibility, stress responsivity, and social integration.^14^ The less consistent findings for neurotic disorders, however, suggests some nuances in the causal mechanisms activated. Neurotic disorders (F40-F48) comprise phobic disorders, obsessive compulsive disorders, posttraumatic stress and adjustment disorders, dissociative disorders, and somatoform disorders, among others. Notably, many of these neurotic disorders are more strongly driven by specific vulnerabilities and event-contingent triggers, including conditioning experiences, trauma exposure, and disorder-specific cognitive-behavioral mechanisms, while mood disorders tend to be cumulative, socially patterned, and mediated through modifiable processes such as affect regulation and stress exposure^29^; this suggests that arts participation and cultural engagement are associated with decreased risk of conditions with an etiological structure that is more gradual and cumulative than rapid-onset, event-contingent disorders.

Further, while the results for dementia were consistent across most models, they were no longer significant in the Fine-Gray model. This finding suggests that cultural engagement may be associated with a longer time to dementia in people who remain alive, but because cultural engagement is also associated with longer lifespan, this does not necessarily translate into a lower cumulative incidence once death is treated as a competing risk. However, it is important to note that this finding was different to previous results in the same cohort that used an algorithm combining self-reported or informant-reported physician diagnosis of dementia or Alzheimer disease or an informant-reported score above the threshold of 3.38 on the 16-question Informant Questionnaire on Cognitive Decline in the Elderly.^30^ Therefore, when dementia is identified not just through hospital diagnoses but also through cognitive batteries, there may still be advantages for individuals who are more cultural engaged. Prodromal effects remain a consideration for dementia, even when excluding cases shortly after baseline, because prodromal symptoms of dementia can influence behaviors long in advance of diagnoses. However, this analysis provided the longest time to follow-up date (20 years). Replication of the analyses again at 25 and 30 years as the cohort continues will provide further insight.

Cultural engagement is a socially patterned behavior, and concerns about residual confounding have been central to the interpretation of previous findings. Methodologically, these findings extend prior work by applying a more rigorous triangulation framework, including utilizing negative outcome controls for the first time.^17^ Negative controls cannot eliminate all residual confounding, but they do add specificity; if cultural engagement were simply a proxy for generalized privilege, health-seeking, or unmeasured environmental advantage, one might expect more diffuse protective associations across socially patterned outcomes. However, the absence of associations with eye disease, ear disease, and traumatic brain injury provides evidence of specificity that is not easily explained by generalized health selection or structural advantage; this suggests that cultural engagement is unlikely to be merely a proxy for broader socioeconomic or behavioral profiles. Additionally, the extended follow-up period and exclusion of early cases add further reassuring evidence to longstanding concerns regarding prodromal bias. While no single method can establish causality in observational research, the convergence of evidence across these approaches strengthens confidence that the observed associations are not solely artifactual. These findings have important public health implications because they raise the question of whether promoting participation in cultural activities through community-based programs and social prescribing may help reduce the risk of clinically diagnosed mental disorders and dementia at the population level. Future research is therefore encouraged, taking experimental approaches to extend the findings presented here.

### Limitations

This study has limitations. First, cultural engagement was self-reported and measured at a single time point, focusing on only a subset of overall cultural behaviors. As a result, it presents an important insight into how certain cultural behaviors are associated with mental disorders. However, future work that considers broader definitions and examines the impact of changes in engagement patterns over time is encouraged. Moreover, different (albeit comparatively similar) cultural experiences were grouped into the same category preventing separate analyses of their associations. Future studies disentangling whether certain patterns of active ingredients within cultural behaviors are particularly important are recommended. Second, although extensive confounders were included, residual confounding cannot be entirely excluded, particularly from unmeasured variables such as personality traits or early-life exposures. While E-value analyses suggest that substantial unmeasured confounding would be required to fully explain the findings, and previous fixed-effects analyses have indicated that unmeasured time-invariant confounders have little influence on models,^31^ it is important to continue probing confounding. Third, the sample was predominantly White and older, which may limit generalizability to more diverse populations or younger age groups. Fourth, the use of hospital-treated outcomes provides important objective data on more severe experiences that warranted hospital mental health care. However, only a minority of cases reach hospital, and many remain undiagnosed entirely. Therefore, it remains important to interpret findings alongside studies that leverage symptom-based scales too. Future research is recommended that explores diagnoses within Mental Health Services datasets when data linkages can be undertaken.

## Conclusions

This cohort study of 8274 adults found that frequent cultural engagement was associated with lower risk of mental disorders overall, as well as specific disorders including dementia, substance misuse, and mood disorders. This study strengthens the case for cultural engagement as a potentially important population-level determinant of mental health. Its main contribution is not simply to replicate prior associations, but to show that these associations are detectable for clinically recorded hospital outcomes, span multiple psychiatric domains, and remain after a set of methodological tests specifically designed to challenge common noncausal explanations. In that respect, the findings help move the field from descriptive optimism toward a more mature causal epidemiology of arts and mental health.

## Notes

Supplement 1. eFigure 1. Participants selection eMethods. eTable 1. Included ICD-10 chapters and codes eFigure 2. Directed acyclic graph illustrating the assumed causal relationships between cultural engagement, potential confounders, and mental health outcomes eTable 2. Case numbers by ICD-10 code eFigure 3. Panel of regression of baseline characteristics predicting each outcome eTable 3. Association between cultural engagement and mental disorders excluding diseases that developed shortly after baseline eTable 4. Association between cultural engagement and mental disorders further adjusting for (i) cognition and depression at baseline and (ii) negative control outcomes (traumatic brain injury, and eye and ear diseases) eTable 5. Association between cultural engagement and mental disorders using tertiles of cultural engagement (range 0-15) eTable 6. E value to consider the unmeasured confounding effect eTable 7. Association between cultural engagement and mental disorders when treating wave 2 as the baseline eTable 8. Association between cultural engagement and mental disorders using the Fine–Gray subdistribution hazards model eTable 9. Association between cultural engagement and mental disorders using the survey-weighted Cox proportional hazards regression models

Supplement 2. Data sharing statement
