# Supplementary material for: Cultural Engagement and Hospital-Treated Mental Disorders in Older Adults
Source: JAMA Netw Open. 2026 Oct 14;9(10):e2639041. doi: 10.1001/jamanetworkopen.2026.39041 (PMC13670060; doi:10.1001/jamanetworkopen.2026.39041)
Supplement: Supplementary file 1 [file jamanetwopen-e2639041-s001.pdf]

Supplemental Online Content

Qin P, Steptoe A, Fancourt D. Cultural engagement and hospital-treated mental disorders in older adults. *JAMA Netw. Open*. 2026;9(10):e2639041. doi:10.1001/jamanetworkopen.2026.39041

eFigure 1. Participants selection
eMethods.
eTable 1. Included *ICD-10* chapters and codes .
eFigure 2. Directed acyclic graph illustrating the assumed causal relationships between cultural engagement, potential confounders, and mental health outcomes
eTable 2. Case numbers by *ICD-10* code
eFigure 3. Panel of regression of baseline characteristics predicting each outcome
eTable 3. Association between cultural engagement and mental disorders excluding diseases that developed shortly after baseline
eTable 4. Association between cultural engagement and mental disorders further adjusting for (i) cognition and depression at baseline and (ii) negative control outcomes (traumatic brain injury, and eye and ear diseases)
eTable 5. Association between cultural engagement and mental disorders using tertiles of cultural engagement (range 0-15)
eTable 6. E value to consider the unmeasured confounding effect
eTable 7. Association between cultural engagement and mental disorders when treating wave 2 as the baseline
eTable 8. Association between cultural engagement and mental disorders using the Fine–Gray subdistribution hazards model
eTable 9. Association between cultural engagement and mental disorders using the survey-weighted Cox proportional hazards regression models

This supplemental material has been provided by the authors to give readers additional information about their work.

© 2026 Qin P et al. *JAMA Network Open*.

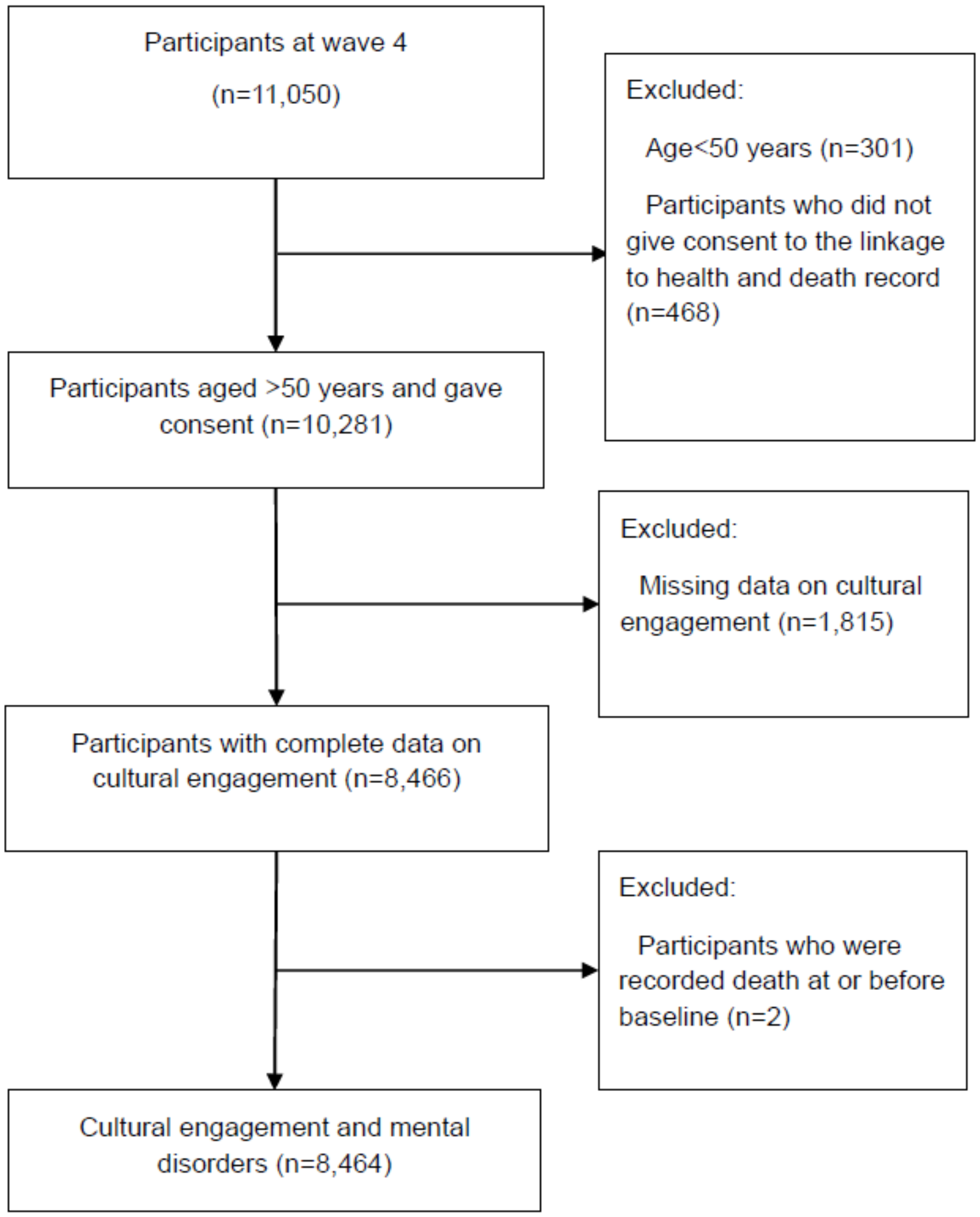

eFigure 1. Participants selection

© 2026 Qin P et al. *JAMA Network Open*.

eMethods

Measurement of outcomes

ELSA participants who had given consent were linked by unique identification number to the National Health Service's Central Registry for vital status data up to November 2024, Hospital Episode Statistics (HES) Admitted Patient Care (HES APC) data, and HES Outpatient (HES OP) data up to March 2024. Linked health registry data provided data for 11 years prior to the study's baseline, meaning any conditions diagnosed in this 11-year pre-baseline period were excluded.

Covariates

Sociodemographic variables, self-reported by participants, included age (in years), sex (male or female), educational qualifications (No qualifications, basic qualifications, high school qualifications, or college degree or higher), ethnicity (White or non-White), total net non-housing wealth (categorised in 5 quintiles), marital status (married/cohabiting or not), employment status (fulltime, part-time, not in employment), and occupational status (managerial and professional occupations, intermediate occupations, small employers and self employed, lower supervisory and technical occupations, and semi-routine occupations; categorised using the five item National Statistics Socio-economic Classification). We used data provided in the nearest subsequent waves if they were missing at baseline.

Lifestyle confounders included smoking status (never, ever and current smokers), frequency of alcohol consumption (>=5 days/week or not), and physical activity level. Physical activity level was categorized into three groups: vigorous (engaging in vigorous activity ≥once a week), moderate (engaging in moderate activity ≥once a week), and inactive (all others). Social covariates included whether living alone (yes or no), being social isolated (yes or no), and being involved with no other community activities (yes or no). Social isolation was measured as whether participants had low contact (less than monthly contact via phone, email or face-to-face) with any social ties (friends, children and wider relatives). Community activities were defined as involvement in any community activities, including being a member of a political party or environmental group, a tenants or neighbourhood watch group, a church or religious association, a charitable association, an education, a social club, a sports, gym or exercise class, or any other society. Across our analyses, model 0 adjusted for age and sex, while model 1 additionally adjusted for ethnicity, educational

© 2026 Qin P et al. *JAMA Network Open*.

qualifications, wealth, marital status, job, and employment. Cultural engagement is a behaviour that can covary with other lifestyle factors and health-related behaviours. However, it is complex to ascertain the directionality of this covariation, leading to challenges in determining when models are under- vs over-adjusted. Consequently, we did undertake further model adjustment, but this was in two additional models to enable a comparison of findings with and without these lifestyle factors. Model 2 additionally adjusted for smoking status, alcohol consumption, physical activity level. Model 3 additionally adjusted for social factors, including living alone, whether being social isolated, and whether reporting no community activities. eFigure 2 shows the directed acyclic graph illustrating the assumed causal relationships between cultural engagement, potential confounders, and mental health outcomes.

In sensitivity analyses we explored further factors that could influence morbidity (particularly if indicative of prodromal effects of imminent morbidity on cultural behaviours) but are known to lie on the causal pathway. The 8-item version of the Center for Epidemiologic Studies Depression scale (CES-D) was used to measure depression, with the score split into highest quartile vs other quartiles. Memory function was measured by summing scores on immediate and delayed memory (ranging from 0 to 20), while executive function was assessed using an animal naming test (semantic verbal fluency), where participants were asked to list as many animals as possible within one minute. Higher overall scores reflect better memory and executive function, and as for depression the score was split into lowest quartile indicating poorest cognitive functioning vs other quartiles.

Sensitivity analyses

To assess the robustness of the observed association to unmeasured confounding, we calculated E-values, which quantify the minimum strength of association that an unmeasured confounder would need with both the exposure and the outcome to fully explain away an observed association, conditional on the measured covariates [24]. To test whether results were heavily influenced by reverse causality, such as people with imminent diagnoses already experiencing prodromal symptoms that could influence their cultural engagement, we undertook further analyses excluding participants who had incident disease within 2 years. We also ran a further analysis for overall mental disorders additionally adjusting for baseline depression and cognition depression and cognition at baseline and for all mental outcomes additionally adjusting for baseline negative control outcomes. To consider whether the index that we had created of cultural engagement may be biased to mis-represent patterns of cultural behaviour, we calculated the index in an alternative way that simultaneously considered both frequency and diversity of engagement. To explore potential

© 2026 Qin P et al. *JAMA Network Open*.

dose-response relationship reported by our previous study [25] and possible measurement error, frequency of cultural engagement was treated as a continuous variable [25]. To check the robustness of the findings in longer follow-up, wave 2 were treated as baseline, with up to 20 years of follow-up. Finally, to consider robustness to alternative statistical choices, competing-risk regression was performed using the Fine–Gray subdistribution hazards model, treating death before the occurrence of the outcome as the competing event. The uses a propensity model based on well-known predictor variables for dementia to estimate the probability that individuals had dementia so that deaths with a high probability of having dementia are reclassified as the main event and deaths with a low probability of dementia are maintained as the competing risk event, as described elsewhere [26]. We also performed survey-weighted Cox proportional hazards regression models fitted using the relevant ELSA sampling weights to account for the complex sampling design and differential non-response.

© 2026 Qin P et al. *JAMA Network Open*.

eTable 1. Included ICD-10 chapters and codes .

Category	Disease	ICD-10 code
**Mental disorders**	**Mental disorders**	F00–F99
	Dementia	F00–F03, G30, G31
	Substance abuse	F10–F19
	Psychotic disorders	F20–F29
	Mood disorders	F30–F39
	Neurotic disorders	F40–F48
**Negative control diseases**	**Diseases of the eye**	H00–H59
	**Diseases of the ear**	H60–H99
	**Traumatic brain injury**	S2, S6, S71, S78, S79, S97, S98, S99, T04, T06

© 2026 Qin P et al. *JAMA Network Open*.

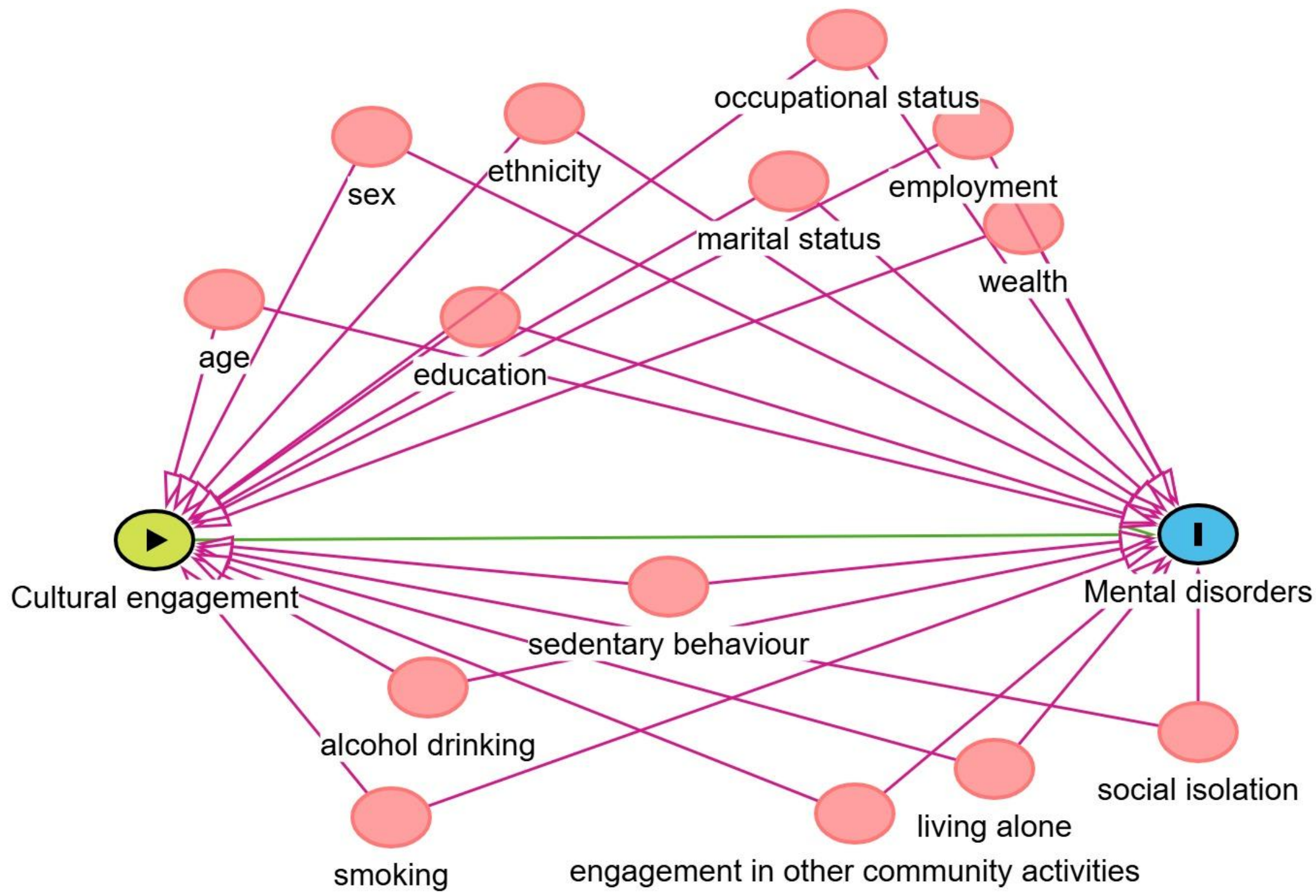

eFigure 2. Directed acyclic graph illustrating the assumed causal relationships between cultural engagement, potential confounders, and mental health outcomes

© 2026 Qin P et al. *JAMA Network Open*.

eTable 2. Case numbers by ICD-10 code

Disease	N	Cases	Follow-up duration Mean (SD)	Incidence (%)	Incidence rate (per 1000 person-year)
**Mental disorders**	8274	2258	11.97 (4.66)	28.14	22.81
Dementia	8454	750	12.98 (4.12)	8.98	6.83
Substance misuse	8396	790	12.63 (4.37)	9.73	7.45
Mood disorders	8389	655	12.78 (4.24)	8.23	6.11
Neurotic disorders	8416	560	12.86 (4.13)	6.94	5.17
**Negative control diseases**					
Eye diseases	7653	2259	12.95 (4.15)	29.51	25.18
Ear diseases	8419	92	13.10 (4.04)	1.09	0.83
Traumatic brain injury	8367	386	11.72 (4.77)	4.61	3.56

© 2026 Qin P et al. *JAMA Network Open*.

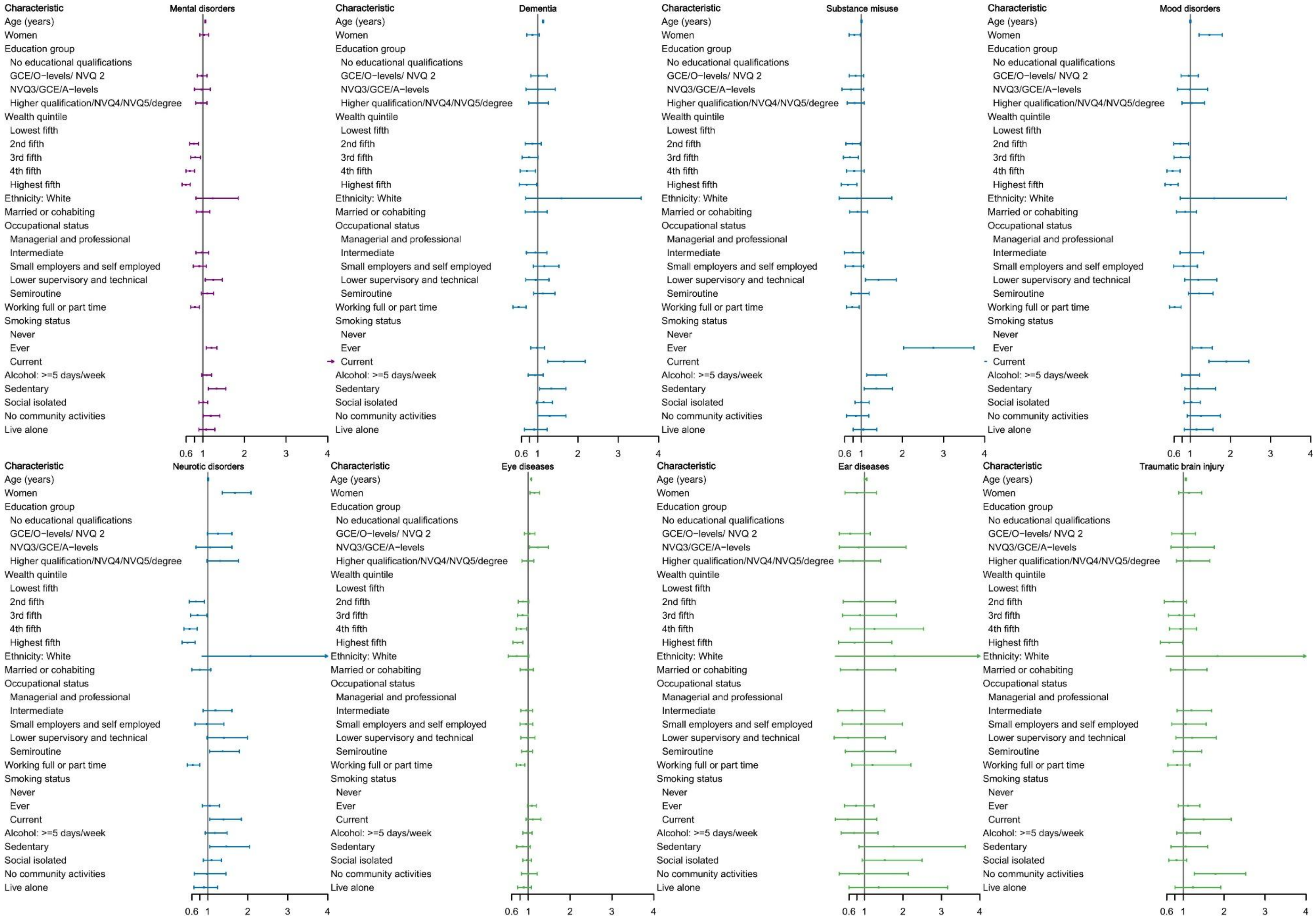

eFigure 3 panel of regression of baseline characteristics predicting each outcome

© 2026 Qin P et al. *JAMA Network Open*.

eTable 3. Association between cultural engagement and mental disorders excluding diseases that developed shortly after baseline

	**Excluding diseases that developed in two years**		
	**HR (95% CI)**	**P value**	**FDR adjusted P value**
**Mental disorders**			
Infrequent	0.77 (0.68–0.87)	**<0.001**	**<0.001**
Frequent	0.72 (0.63–0.83)	**<0.001**	**<0.001**
**Dementia**			
Infrequent	0.78 (0.63–0.95)	**0.015**	**0.035**
Frequent	0.70 (0.56–0.89)	**0.003**	**0.009**
**Substance misuse**			
Infrequent	0.77 (0.63–0.95)	**0.012**	**0.029**
Frequent	0.76 (0.60–0.96)	**0.023**	**0.047**
**Mood disorders**			
Infrequent	0.74 (0.58–0.95)	**0.017**	**0.036**
Frequent	0.73 (0.56–0.96)	**0.023**	**0.047**
**Neurotic disorders**			
Infrequent	0.89 (0.68–1.17)	0.417	0.485
Frequent	0.87 (0.65–1.17)	0.361	0.428
**Negative control diseases**			
**Eye diseases**			
Infrequent	0.96 (0.83–1.11)	0.568	0.606
Frequent	1.03 (0.88–1.20)	0.709	0.71
**Ear diseases**			
Infrequent	0.88 (0.46–1.68)	0.689	0.710
Frequent	0.69 (0.33–1.43)	0.313	0.409
**Traumatic brain injury**			
Infrequent	0.71 (0.52–0.98)	0.035	0.065
Frequent	0.79 (0.56–1.11)	0.171	0.257

Model adjusted for age and gender, ethnicity, education, marital status, wealth, employment, occupational status, smoking, alcohol drinking, and sedentary behaviour, living alone, social isolation and engagement in other community activities).

© 2026 Qin P et al. *JAMA Network Open*.

eTable 4. Association between cultural engagement and mental disorders further adjusting for (i) cognition and depression at baseline and (ii) negative control outcomes (traumatic brain injury, and eye and ear diseases)

	HR (95% CI)	p-value
**(i) Cultural engagement**		
Never	ref	
Infrequent	0.83 (0.74-0.94)	**0.003**
Frequent	0.79 (0.68, 0.90)	**<0.001**
**(ii) Cultural engagement**		
Never	ref	
Infrequent	0.72 (0.91, 0)	**0.005**
Frequent	0.65 (0.85, 0)	**<0.001**

Model adjusted for age, gender, ethnicity, education, marital status, wealth, employment, occupational status, smoking, alcohol drinking, sedentary behaviour, living alone, social isolation and engagement in other community activities, memory (lowest quartiles vs. others) and executive function (lowest quartiles vs. others) and depression (highest quartiles vs. others) at baseline.

eTable 5. Association between cultural engagement and mental disorders using tertiles of cultural engagement (range 0-15)

	Full adjustment model (tertile 1 as reference group)		
**Diseases by category**	**HR (95% CI)**	**P value**	**FDR adjusted P value**
**Mental disorders**			
Tertile 2	0.73 (0.65–0.82)	**<0.001**	**<0.001**
Tertile 3	0.73 (0.64–0.83)	**<0.001**	**<0.001**
**Dementia**			
Tertile 2	0.76 (0.62–0.93)	**0.007**	**0.017**
Tertile 3	0.82 (0.66–1.03)	0.090	0.134
**Substance misuse**			
Tertile 2	0.76 (0.63–0.92)	**0.005**	**0.013**
Tertile 3	0.73 (0.58–0.93)	**0.011**	**0.023**
**Mood disorders**			
Tertile 2	0.72 (0.58–0.89)	**0.003**	**0.007**
Tertile 3	0.73 (0.57–0.93)	**0.010**	**0.023**
**Neurotic disorders**			
Tertile 2	0.81 (0.64–1.02)	0.070	0.112
Tertile 3	0.79 (0.61–1.03)	0.079	0.121
**Negative control diseases**			
**Eye diseases**	0.93 (0.83–1.05)		
Tertile 2	1.06 (0.94–1.21)	0.237	0.31
Tertile 3		0.341	0.404
**Ear diseases**	0.85 (0.47–1.53)		
Tertile 2	0.90 (0.47–1.72)	0.591	0.61
Tertile 3		0.749	0.749
**Traumatic brain injury**			

© 2026 Qin P et al. *JAMA Network Open*.

Tertile 2	0.80 (0.60–1.06)	0.12	0.164
Tertile 3	0.89 (0.66–1.21)	0.462	0.518

Model adjusted for age, gender, ethnicity, education, marital status, wealth, employment, occupational status, smoking, alcohol drinking, sedentary behaviour, living alone, social isolation and engagement in other community activities.

eTable 6. E value to consider the unmeasured confounding effect

Diseases	E-value point estimate (CI)			
	**Model 0**	**Model 1**	**Model 2**	**Model 3**
**Mental disorders**				
Infrequent	2.16 (1.97)	1.82 (1.58)	1.66 (1.41)	1.69 (1.44)
Frequent	2.70 (2.47)	2.16 (1.91)	1.79 (1.53)	1.85 (1.56)
**Dementia**				
Infrequent	2.21 (1.70)	2.08 (1.53)	2.00 (1.39)	1.85 (1.21)
Frequent	2.66 (2.04)	2.45 (1.77)	2.26 (1.60)	2.17 (1.50)
**Substance misuse**				
Infrequent	3.97 (3.52)	2.55 (1.96)	1.92 (1.36)	1.92 (1.36)
Frequent	6.60 (5.33)	3.77 (2.90)	1.88 (1.16)	2.00 (1.28)
**Mood disorders**				
Infrequent	2.61 (2.00)	1.96 (1.32)	1.81 (1.11)	1.85 (1.00)
Frequent	3.50 (2.66)	2.26 (1.53)	1.96 (1.21)	2.08 (1.29)
**Neurotic disorders**				
Infrequent	2.40 (1.74)	1.85 (1.00)	1.67 (1.00)	1.67 (1.00)
Frequent	3.04 (2.26)	2.00 (1.16)	1.81 (1.00)	1.77 (1.00)

This eTable showed the E value of the risk estimates compared to the never engaged group.
Model 0 adjusted for age and gender.
Model 1 further adjusted for socio-demographic and economic factors (ethnicity, education, marital status, wealth, employment, and occupational status).
Model 2 further adjusted for lifestyle factors (smoking, alcohol drinking, and sedentary behaviour).
Model 3 further adjusted for social covariates (living alone, social isolation and engagement in other community activities).

© 2026 Qin P et al. *JAMA Network Open*.

eTable 7. Association between cultural engagement and mental disorders when treating wave 2 as the baseline

	Full adjustment model (never engaged as reference group)		
**Diseases by category**	**HR (95% CI)**	**P value**	**FDR adjusted P value**
**Mental disorders**			
Infrequent	0.77 (0.67–0.87)	**<0.001**	**<0.001**
Frequent	0.70 (0.60–0.81)	**<0.001**	**<0.001**
**Dementia**			
Infrequent	0.85 (0.69–1.04)	0.104	0.196
Frequent	0.77 (0.62–0.97)	**0.027**	0.063
**Substance misuse**			
Infrequent	0.61 (0.48–0.77)	**<0.001**	**<0.001**
Frequent	0.66 (0.51–0.86)	**0.002**	**0.007**
**Mood disorders**			
Infrequent	0.98 (0.75–1.27)	0.853	0.88
Frequent	0.88 (0.66–1.18)	0.386	0.494
**Neurotic disorders**			
Infrequent	0.72 (0.54–0.96)	**0.023**	0.055
Frequent	0.58 (0.42–0.80)	**0.001**	**0.003**
**Negative control diseases**			
**Eye diseases**			
Infrequent	0.91 (0.80–1.04)	0.155	0.283
Frequent	0.91 (0.79–1.04)	0.172	0.307
**Ear diseases**			
Infrequent	1.02 (0.51–2.03)	0.959	0.97
Frequent	0.91 (0.43–1.92)	0.798	0.852
**Traumatic brain injury**			
Infrequent	1.06 (0.76–1.49)	0.734	0.82
Frequent	0.94 (0.65–1.36)	0.749	0.82

Model adjusted for age, gender, ethnicity, education, marital status, wealth, employment, occupational status, smoking, alcohol drinking, sedentary behaviour, living alone, social isolation and engagement in other community activities.

eTable 8. Association between cultural engagement and mental disorders using the Fine–Gray subdistribution hazards model

	**HR (95% CI)**	**P value**	**FDR adjusted P value**
**Mental disorders**			
Infrequent	0.84 (0.75, 0.95)	**0.007**	0.056
Frequent	0.8 (0.69, 0.91)	**0.001**	0.016
**Dementia**			
Infrequent	0.96 (0.78, 1.18)	0.70	0.789
Frequent	0.92 (0.72, 1.16)	0.46	0.684
**Substance misuse**			
Infrequent	0.78 (0.65, 0.95)	**0.013**	0.069
Frequent	0.79 (0.62, 1.00)	**0.046**	0.147
**Mood disorders**			
Infrequent	0.83 (0.66, 1.03)	0.096	0.192
Frequent	0.77 (0.60, 0.99)	**0.04**	0.147
**Neurotic disorders**			
Infrequent	0.96 (0.74, 1.24)	0.74	0.789
Frequent	0.9 (0.67, 1.20)	0.47	0.684
**Negative control diseases**			
**Eye diseases**			
Infrequent	0.9 (0.81, 1.01)	0.086	0.192
Frequent	0.97 (0.85, 1.09)	0.58	0.763
**Ear diseases**			
Infrequent	0.95 (0.56, 1.61)	0.85	0.85
Frequent	0.87 (0.50, 1.52)	0.62	0.763
**Traumatic brain injury**			
Infrequent	0.8 (0.62, 1.04)	0.093	0.192
Frequent	0.82 (0.62, 1.09)	0.17	0.302

© 2026 Qin P et al. *JAMA Network Open*.

eTable 9. Association between cultural engagement and mental disorders using the survey-weighted Cox proportional hazards regression models

	HR (95% CI)	P value	FDR adjusted P value
**Mental disorders**			
Infrequent	0.83 (0.71–0.96)	**0.014**	**0.042**
Frequent	0.73 (0.62–0.87)	**<0.001**	**0.001**
**Dementia**			
Infrequent	0.73 (0.57–0.93)	**0.01**	**0.033**
Frequent	0.68 (0.52–0.90)	**0.007**	**0.033**
**Substance misuse**			
Infrequent	0.89 (0.67–1.18)	0.412	0.515
Frequent	0.95 (0.68–1.34)	0.777	0.777
**Mood disorders**			
Infrequent	0.78 (0.59–1.03)	0.082	0.137
Frequent	0.71 (0.52–0.97)	**0.031**	0.062
**Neurotic disorders**			
Infrequent	0.90 (0.65–1.26)	0.549	0.610
Frequent	0.85 (0.59–1.22)	0.381	0.515

© 2026 Qin P et al. *JAMA Network Open*.
