# Supplementary material for: Cultural Engagement and Hospital-Treated Mental Disorders in Older Adults
Source: JAMA Netw Open. 2026 Oct 14;9(10):e2639041. doi: 10.1001/jamanetworkopen.2026.39041 (PMC13670060; doi:10.1001/jamanetworkopen.2026.39041)
Supplement: Supplementary file 2 [file jamanetwopen-e2639041-s002.pdf]

Data Sharing Statement

Qin. Cultural Engagement and Hospital-Treated Mental Disorders in Older Adults. *JAMA Netw Open*. Published October 14, 2026. doi:10.1001/jamanetworkopen.2026.39041

Data

Data available: Data sharing statement ELSA data are available through registration with the UK data service (https://beta.ukdataservice.ac.uk/datacatalogue/series/series?id=200011). The linked HES and mortality data cannot be shared directly due to data sharing agreements with NHS England, but applications for data access can be made via the UK Longitudinal Linkage Collaboration: https://ukllc.ac.uk/apply.
